# Pharmacogenetic Testing for Prevention of Severe Cutaneous Adverse Drug Reactions

**DOI:** 10.3389/fphar.2020.00969

**Published:** 2020-07-02

**Authors:** Chih-Jung Chang, Chun-Bing Chen, Shuen-Iu Hung, Chao Ji, Wen-Hung Chung

**Affiliations:** ^1^ Department of Dermatology and Drug Hypersensitivity Clinical and Research Center, Chang Gung Memorial Hospital, Linkou, Taipei and Keelung, Taiwan; ^2^ Central Research Laboratory, Department of Dermatology and Xiamen Chang Gung Allergology Consortium, Xiamen Chang Gung Hospital, School of Medicine, Huaqiao University, Xiamen, China; ^3^ Cancer Vaccine and Immune Cell Therapy Core Laboratory, Department of Medical Research, Chang Gung Memorial Hospital, Taoyuan, Taiwan; ^4^ College of Medicine, Chang Gung University, Taoyuan, Taiwan; ^5^ Whole-Genome Research Core Laboratory of Human Diseases, Chang Gung Memorial Hospital, Keelung, Taiwan; ^6^ Graduate Institute of Clinical Medical Sciences, Chang Gung University, Taoyuan, Taiwan; ^7^ Department of Dermatology, The First Affiliated Hospital of Fujian Medical University, Fuzhou, China; ^8^ Department of Dermatology, Beijing Tsinghua Chang Gung Hospital, School of Clinical Medicine, Tsinghua University, Beijing, China; ^9^ School of Medicine, Shanghai Jiao Tong University, Shanghai, China

**Keywords:** drug hypersensitivity, pharmacogenetics, severe cutaneous adverse reactions, human-leukocyte antigen, T cell receptor

## Abstract

Severe cutaneous adverse reactions (SCAR), such as Stevens-Johnson syndrome (SJS), toxic epidermal necrolysis (TEN), and drug rash with eosinophilia and systemic symptoms (DRESS), are idiosyncratic and unpredictable drug-hypersensitivity reactions with a high-mortality rate ranging from 10% to over 30%, thus causing a major burden on the healthcare system. Recent pharmacogenomic studies have revealed strong associations between SCAR and the genes encoding human-leukocyte antigens (HLAs) or drug-metabolizing enzymes. Some of pharmacogenetic markers have been successfully applied in clinical practice to protect patients from SCAR, such as HLA-B*15:02 and HLA-A*31:01 for new users of carbamazepine, HLA-B*58:01 for allopurinol, and HLA-B*57:01 for abacavir. This article aims to update the current knowledge in the field of pharmacogenomics of drug hypersensitivities or SCAR, and its implementation in the clinical practice.

## Introduction

**Figure 1:**
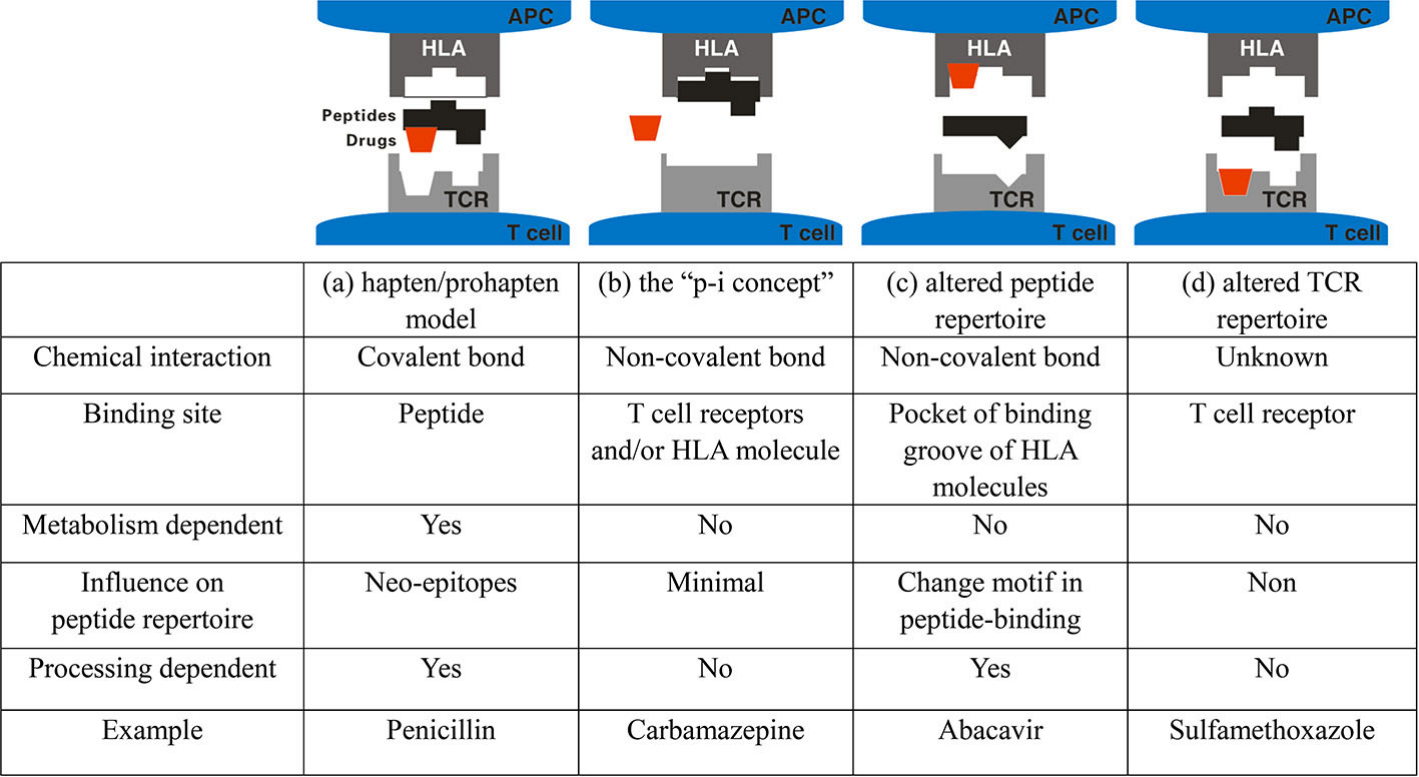
Four models of interaction between small molecule drugs and the HLA-peptide-TCR in drug hypersensitivity. **(A)** Hapten/pro-hapten concept: Drugs or reactive metabolites serve as haptens and bind to the endogenous peptides to form the haptenated peptides (neo-epitopes) that presented by HLA molecules at the cell surface where the novel peptide is recognized as foreign. The HLA/drug/peptide complex is recognized by TCR, which trigger the drug-specific T cell activation. **(B)** The “pharmacological interaction with immune receptors (p-i)” concept: T cells are proposed to recognize immunogenic complexes formed through a labile interaction of the causative drug, HLA and TCR at the cell surface or TCR and active drug-specific T cells without the intercellular processing in APC. **(C)** The altered repertoire model: The drug interacts with the antigen-binding cleft of the HLA molecule altering the space available to anchor residues of peptide ligands and results in selection of ligands with a novel HLA binding motif, which promotes polyclonal T-cell activation. **(D)** The “altered TCR repertoire” model. Drugs bind to TCR, resulting in conformational change of TCR, which then bind to the HLA/self-peptide complex to elicit immune reaction.

Some drugs can induce inappropriate hypersensitivity/immune response ranging from milder forms, such as urticarial and maculopapular exanthema (MPE), to more severe clinical presentations such as acute generalized exanthematous pustulosis (AGEP), Stevens-Johnson syndrome (SJS), toxic epidermal necrolysis (TEN), and drug rash with eosinophilia and systemic symptoms (DRESS). All these conditions are classified as severe cutaneous adverse drug reactions (SCAR). The immune mechanism of SCAR is classified as type-IV hypersensitivity reaction characterized by the activation of lymphocytes such as CD4^+^ and CD8^+^ T cells. Type -IV hypersensitivity is also called delayed-type hypersensitivity and often develops few days or weeks after drug exposure (Pichler, 2003). Although their incidence is low, SCAR are usually unpredictable and potentially lethal events (Wolf et al., 2005). AGEP is a rare, acute eruption that presents with numerous non-follicular sterile pustules in the epidermis. Fever and peripheral-blood leukocytosis are usually present in patients with AGEP. Approximately 90% of AGEP cases are induced by systemic drugs, particularly antibiotics such as aminopenicillins, macrolides, and antifungals (Roujeau et al., 1991). In comparison, SJS/TEN, including SJS, SJS-TEN overlap, and TEN, are on the same disease spectrum but with different severity and the extent of skin detachment. All these usually present as patches, atypical targetoid macules, and erythematous or violaceous macules in skin lesions. In addition, mucocutaneous involvement is a common feature that develops in the oral mucosa of patients with SJS/TEN, with a few incidences in ocular, genital, or anal mucosa. This is classified by the degree of detachment over the total body-surface area; less than 10% is considered SJS, 10–30% is considered as SJS-TEN overlap, and over 30% is considered as TEN (Lee and Chung, 2013). This feature differentiates SJS/TEN from SJS and TEN. In DRESS, there is less or no skin detachment and mucocutaneous involvement present, with maculopapular exanthema being the most common presentation. Significant multisystem involvement is common and may include hematologic, hepatic, renal, pulmonary, cardiac, neurological, gastrointestinal, and endocrine abnormalities (Husain et al., 2013). Many studies have revealed that interactions between human leukocyte antigen (HLA) and drugs are critical for the induction of T lymphocyte activity *via* T cell receptor (TCR) in patients with SCAR (Bharadwaj et al., 2012; Camous et al., 2012; Hoetzenecker et al., 2016). Generally, two main types of HLA molecules are reported to be presented: HLA class I molecules and HLA class II molecules, which are expressed on most nucleated cells and antigen-presenting cells (APCs), such as monocytes or dendritic cells (DCs), respectively. HLA-A, HLA-B, and HLA-C belong to HLA class I molecules. HLA-DR, HLA-DQ, and HLA-DP belong to HLA class II molecules. Adaptive activation of CD8^+^ and CD4^+^ T lymphocytes is initiated by antigen presentation. Overall, four mechanistic hypotheses have been proposed to explain how small compounds are recognized by T cells in an HLA-dependent manner: i) the “hapten/prohapten” theory; ii) the “p-i concept”; iii) the “altered peptide repertoire” model; and iv) the “altered TCR repertoire” model have (**Figure 1**) (Yun et al., 2016; Chung et al., 2016). In the hapten/prohapten theory, culprit drugs or the reactive metabolites are too small to elicit immunogenicity, whereas they act as haptens interact covalently with endogenous peptides/proteins to form a drug/haptenated peptides complex in the host. The drug, self-peptides, and HLA molecules form complex by covalent bonds and further resulting in induction of drug-specific immune responses. For example, penicillin binds to endogenous peptides and its presentation by HLA through the classical processing-required pathway to trigger T cell activation resulting in penicillin allergy (Padovan et al., 1997). The “pharmacological interaction with immune receptors (p-i)” concept postulates that drugs or metabolites may directly binds to the HLA and/or TCR protein by unstable and noncovalent interactions independent of the classic antigen-processing pathway in antigen-presenting cells (APCs). For example, carbamazepine (CBZ)/aromatic antiepileptic drugs can directly interact with HLAB* 15:02 in CBZ-SJS/TEN. No intracellular antigen processing or drug metabolism was involved in the HLA-B*15:02 presentation of CBZ, and the appropriate endogenous peptides loading on HLA-B*15:02 was required for the stability of the HLA complex on the cell surface to present CBZ to T cells (Wei et al., 2012). The “altered peptide repertoire” model presents that the culprit drugs occupy the position in the peptide-binding groove of the HLA protein, changing the binding cleft and the peptide specificity of HLA binding. In this model, the peptide presented in the HLA proteins is recognized as the “initial antigen” by immune system, thus triggering a T cell response. The well-known example is that abacavir interacted with F-pocket HLA-B*57:01 and altered shape and chemistry of the antigen-binding cleft, such as tryptophan (W) is replaced with isoleucine (I) or leucine (L) at the C-terminus of repertoire of endogenous peptides. Changed endogenous peptides resulting in polyclonal T cell activation and autoimmune-like systemic reaction manifestations (Illing et al., 2012; Ostrov et al., 2012). This demonstrates a metabolism-independent, direct, noncovalent, and dose dependent association between abacavir and peptides in the HLA-B*57:01 binding cleft. The final hypothesis, recently, Watkins and Pichler proposed a novel “altered TCR repertoire” model that presented a small portion of the drug, such as sulfamethoxazole, alter conformation of TCR regarded as an initial drug interaction that provide a potential bind to HLA/peptides complex and further contribute to the occurrence of SCARs. Various immunological cytokines and cytotoxic proteins are induced to develop skin lesions and subsequently exacerbate the disease. In this review, we highlight the current findings on genetically predisposing markers for the prediction and early diagnosis for SCAR.

## Drug Hypersensitivity and Genetic Susceptibility Markers

Dependent on extended pharmacogenomic studies, we have more advanced understanding on the genetic basis of SCARs. Cases of sulfonamide- and oxicam-related TEN is first report the relationship between HLA alleles and drug-induced SJS/TEN (Roujeau et al., 1987). The biological function of HLAs is to present antigens to the TCR and then elicit specific T cell-dependent immune responses, which is largely correlated with the pathogenesis of SCARs. It has become clear now that HLA associations with SCARs are often drug- and ethnicity-specific. Based on the characteristics, some drugs are restricted to specific HLA in specific populations, whereas some drugs are more general to different HLA alleles and further presented to T cells. Culprit drugs and the HLA alleles associated with SCAR are summarized in **Table 1**. This section deals with the strong association between the reaction to certain drugs and particular HLA alleles that enables predicting hypersensitivity to drugs.

**Table 1:** Human leukocyte antigen (HLA) association between drugs and severe cutaneous adverse reactions (SCARs)/hypersensitivity in different ethnicity.

Drug	HLA allele	SCAR	Ethnicity	Negative predicted value (NPV)	Positive predicted value (PPV)
Abacavir	B*57:01	HSS	Caucasian, Asians (Hetherington et al., 2002; Mallal et al., 2002; Mallal et al., 2008), Black (Saag et al., 2008)	100% in Caucasians	55% in Caucasians
Allopurinol	B*58:01	SJS TEN DRESS	Han Chinese (Hung et al., 2005; Cao et al., 2012) Thai (Tassaneeyakul et al., 2009) Japanese (Kaniwa et al., 2008) Korean (Kang et al., 2011) European (Stamp et al., 2016)	100% in Han Chinese; 100% in Thai	2% in Han Chinese; 1.52% in Thai
Carbamazepine	B*15:02	SJS TEN	Han Chinese (Chung et al., 2004; Man et al., 2007) Thai (Locharernkul et al., 2008; Tangamornsuksan et al., 2013) Indian (Mehta et al., 2009) Malaysian (Chang et al., 2011) Vietnam (Nguyen et al., 2015) Singapore (Chong et al., 2014) Hong Kong (Kwan et al., 2014)	100% in Han Chinese, East Asian	3% in Han Chinese, East Asian
	B*15:11	SJS TEN	Koreans (Kim et al., 2011) Japanese (Kaniwa et al., 2010)		
	B*57:01	SJS TEN	European (Mockenhaupt et al., 2019)	99.98% in European	0.89% in European
	A*31:01	DRESS MPE	Han Chinese (Hung et al., 2006) Korea (Kim et al., 2011) Japanese (Ozeki et al., 2011) Caucasians (Hung et al., 2006; McCormack et al., 2011)	99.97% in Han Chinese	0.59% in Han Chinese
Oxcarbazepine	B*15:02	SJS TEN	Han Chinese (Hung et al., 2010)	99.97% in Han Chinese	0.73% in Han Chinese
Dapsone	B*13:01	DRESS	Han Chinese (Zhang et al., 2013)	99.8% in Han Chinese, East Asian	7.8% in Han Chinese
Phenytoin	B*15:02 B*13:01 B*51:01	SJS TEN	Han Chinese (Hung et al., 2010; Cheung et al., 2013) Thai (Locharernkul et al., 2008) Malaysians (Chang et al., 2017)		
Nevirapine	DRB1*01:01	DRESS	Hispanics, African (Martin et al., 2005)		
Beta-lactam antibiotics	C*04:06, C*08:01, DRB1*04:06	Delayed type hypersensitivity reactions (MPE, DRESS, and SJS	Chinese (Singvijarn et al., 2019)		
Flucloxacillin	B*57:01 B*57:03	DILI	Caucasian (Daly et al., 2009) Britons, Swedish (Nicoletti et al., 2019b)		
Amoxicillin-clavulanate	DRB1*15:01-DRB5*01:01-DQB1*06:02	DILI	Caucasian (Donaldson et al., 2010; Lucena et al., 2011)		

### HLA-Gene Susceptibility for Abacavir Hypersensitivity

Abacavir has the ability to inhibit reverse transcriptase and is used as an adjuvant in combination therapies for the treatment of patients with HIV infection. Approximately 5–7% of patients who receive the drug develop the hypersensitivity syndrome (Hetherington et al., 2001). Most of hypersensitivity patients exhibit at least two symptoms such as fever, rash, vomiting, and gastrointestinal symptoms within 6 weeks after the drug administration, and require immediate medical intervention. Approximately 30% of patients exhibit respiratory symptoms such as dyspnea, cough, and pharyngitis. Re-exposure to abacavir causes rapid exacerbation of symptoms and can deteriorate to more severe conditions. Rare cases of severe reaction induced by abacavir treatment such as DRESS have been reported (Bossi et al., 2002; Pahk et al., 2009). In 2002, studies in two independent populations proposed the association of HLA-B*57:01 as risk factors for abacavir-related hypersensitivity (Hetherington et al., 2002; Mallal et al., 2002) (**Table 1**). HLA-B*57:01 has also been proposed as a genetic marker that indicates abacavir hypersensitivity in white and black participants; 44% of white and 100% of black participants with the HLA-B*57:01 allele experienced abacavir-induced hypersensitivity (Saag et al., 2008) (**Table 1**). In 2008, the largest randomized clinical trial in pharmacogenetics recruited 1,956 patients from 19 countries to confirm HLA-B*57:01 as an effective predictive prescreening for abacavir induced hypersensitivity. The patients were randomly separated into two experimental groups and it was revealed that the incidence of abacavir hypersensitivity was 0% in the screened population, compared with 2.7% in the unscreened-patient population. These results support the use of HLA-B*57:01 as a genetic maker effectively to prevent hypersensitivity caused by abacavir (Mallal et al., 2008) (**Table 1**). A recent study showed that abacavir may occupy the binding groove of HLA, causing alteration of peptide presentation and triggering an autoimmune response (Norcross et al., 2012). Previous crystallography and structure analysis showed a recognition complex that was composed of self-peptide and HLA-B*57:01 (Yerly et al., 2017). Consequently, these results recommended pre-screen for risk genetic factor HLA-B*57:01 before treatment with abacavir (**Table 2**).

**Table 2:** Clinical implementation of pharmacogenomic testing for prevention of SCAR.

Drug	Biomarker	Clinical application	Pharmacogenomic information in drug labeling and guideline
Abacavir	HLA-B*57:01	Application of HLA-B*57:01 testing in clinical practice in Australia, Europe, the US, Thailand, etc.	The US FDA, US HHS, EMA, Canada HCSC, and multiple international HIV/AIDS organizations suggest HLA-B*57:01 genetic testing is required before the first use of abacavir.
Allopurinol	HLA-B*58:01	Application of HLA-B*58:01 testing in clinical practice in Asian countries including Taiwan, Thailand, Korea, and China.	1. The American College of Rheumatology guidelines for the management of gout recommended HLA-B*58:01 testing prior to allopurinol administration. 2. Reimbursement of the testing is supported by national health insurance in China, Taiwan, and Korea. An ongoing project of free test is provided in medical centers in Thailand.
Carbamazepine	HLA-B*15:02	1. Application of HLA-B*15:02 testing in clinical practice in Taiwan, Hong Kong, Singapore, Thailand, etc.	1.The US FDA and Taiwan FDA label HLA-B*15:02 genetic testing is required before the first use of CBZ. 2. Canada HCSC and other drug regulatory agencies such as Thailand HITAP, Hong Kong Department of Health, Singapore Ministry of Health, India MOHFW, and EMA recommend performing HLA-B*15:02 genetic testing before the use of CBZ for patients with certain Asian background. 3. Reimbursement of the testing is supported by national health insurance in Taiwan, China, Hong Kong, Singapore, and Thailand
Carbamazepine	HLA-A*31:01	Ongoing clinical trial of prospective screening of HLA-A*31:01 before prescribing CBZ in Japan	1. US FDA labels the risk of this allele related to CBZ hypersensitivity. 2.Canada HCSC recommends HLA-A*31:01 genetic testing before the use of CBZ in genetically at-risk populations.
Dapsone	HLA-A*31:01	Prospective screening of HLA-B*13:01 before prescribing dapsone in China.	
Oxcarbazepine	HLA-B*15:02	HLA-B*15:02 was found to be significantly associated with OXC-SJS/TEN in Han Chinese and Thai patients.	The US FDA and Taiwan FDA recommend HLA-B*15:02 genetic testing before the use of OXC in patients of Asian ancestry with a high genetic background.
Phenytoin	HLA-B*15:02 and CYP2C9	Ongoing clinical trial prospective screening of CYP2C9*3 with HLA alleles before prescribing PHT in Taiwan	Canada HCSC recommends HLA-B*15:02 genetic testing before the use of PHT.

### HLA-Gene Susceptibility to Aromatic Antiepileptic Drugs-Induced Severe Cutaneous Adverse Reactions

Antiepileptic drugs such as CBZ, phenytoin (PHT), oxcarbazepine (OXC), and lamotrigine (LTG) are used widely to treat epilepsy, bipolar disorder, and trigeminal neuralgia (Marson et al., 2007; Cruccu et al., 2008). Our previous study presented the first report of a strong association of CBZ-induced SJS/TEN with HLA-B*15:02 in Taiwanese patients (Chung et al., 2004). It was observed that all patients with CBZ-induced SJS/TEN carried the HLA-B*15:02 allele, compared with only 3% of drug-tolerant subjects and 8.6% of the general population. Several studies have also shown this association in different populations, specifically in Han Chinese, Thailand, Malaysia, Singapore, India, Vietnam, and Hong Kong (Man et al., 2007; Locharernkul et al., 2008; Mehta et al., 2009; Chang et al., 2011; Tangamornsuksan et al., 2013; Chong et al., 2014; Kwan et al., 2014; Nguyen et al., 2015) (**Table 1**). In 2011, we started a large prospective study with almost 5,000 participants from 23 hospitals in Taiwan to evaluate the benefits of HLA-B*15:02-pretreatment screening. There were 4,120 HLA-B*15:02-negative patients treated with CBZ and 215 HLA-B*15:02-positive individuals treated with an alternative drug. The results showed that SJS/TEN did not develop in any of the CBZ-treated patients without HLA-B*15:02, indicating that the HLA-B*15:02-pretreatment screening could effectively prevent SCAR in comparison with the estimated historical incidence of carbamazepine-induced SJS-TEN (Chen et al., 2011) (**Table 1**). HLA-B*15:02 is ethnically specific in CBZ-induced SJS/TEN because of the genetic background; it is comparatively high in Han Chinese, Malaysian, and Thai populations, compared with Japanese, Korean, and European populations (Alfirevic et al., 2006; Kano et al., 2008; Kaniwa et al., 2010; Chang et al., 2011; Kim et al., 2011; He et al., 2013; Aggarwal et al., 2014) (**Table 1**). In addition to CBZ, the HLA-B*15:02 allele is also used as a risk factor for SCAR induced by other antiepileptic drugs such as OXC (Hung et al., 2010; Chen et al., 2017), PHT (Locharernkul et al., 2008), and LTG (Shi et al., 2011) (**Table 1**). Our previous study provided a detailed molecular mechanism of interaction between HLA and drugs in HLA-associated drug hypersensitivity. The endogenous peptide-loaded HLA-B^*^15:02 molecule presented CBZ to cytotoxic T lymphocytes (CTLs) without the involvement of intracellular drug metabolism or antigen processing. The HLA-B∗15:02/peptide/β(2)-microglobulin protein complex showed binding affinity toward chemicals sharing 5-carboxamide on the tricyclic ring, as with CBZ. There are three amino acid, Asn63, Ile95 as well as Leu156, involved in in CBZ presentation and CTLs activation (Wei et al., 2012), for interaction of HLA molecule and T cells for CBZ. In addition to HLA-B*15:02, HLA-B*15:11 was a potential risk factor for CBZ-induced SJS/TEN in Japanese and Korea patients (Kaniwa et al., 2010; Kim et al., 2011) (**Table 1**). HLA-A*31:01 has been reported as a marker for CBZ hypersensitivity in Europeans (McCormack et al., 2011). A further study showed that HLA-A*31:01 was strongly associated with CBZ-induced DRESS but not SJS*/*TEN in European and Han Chinese populations (Hung et al., 2006; Genin et al., 2014; Nicoletti et al., 2019a) (**Table 1**). Occurring at a low frequency in Japanese, Korean, and European populations, HLA-A*31:01 has been shown to closely correlate with CBZ-induced DRESS by meta-analyses (Kim et al., 2011; McCormack et al., 2011; Ozeki et al., 2011; Genin et al., 2014; Nicoletti et al., 2019a) (**Table 1**). A prospective screening of HLA-A*31:01 in new Japanese CBZ users showed effective prevention of hypersensitivity reactions induced by CBZ (Mushiroda et al., 2018). In addition, a recent study from RegiSCAR group showed HLA-B*57:01 was strongly associated with patients of CBZ-induced SJS/TEN in Europeans (Mockenhaupt et al., 2019) (**Table 1**). HLA-B*59:01 was also reported to be a risk gene for CBZ-induced SJS/TEN (Ikeda et al., 2010). Consequently, genetic pre-screening prior to the use of CBZ in certain Asian populations is recommended (Ferrell and McLeod, 2008) (**Table 2**).

### HLA-Gene Susceptibility to Allopurinol-Induced Severe Cutaneous Adverse Reactions

Allopurinol is commonly used in patients with hyperuricemia-associated disorders such as chronic gout, uric acid nephrolithiasis, and tumor-lysis syndrome *via* xanthine-oxidase inhibition. Approximately 0.1–0.4% of the exposed patients have been reported to develop DRESS- and SJS*/*TEN-hypersensitivity reactions (Hershfield et al., 2013). The incidence of allopurinol-induced SJS*/*TEN was much higher than that induced by aromatic anticonvulsants in Europe but ranked second behind CBZ in southeast Asia (Lee et al., 2014; Cheng et al., 2014). Previously, we recruited 51 patients with allopurinol-induced SJS/TEN, 135 tolerant participants, and 93 healthy subjects. We observed that all patients with allopurinol-induced SJS/TEN had the HLA-B*58:01 allele; in tolerant participants and healthy controls, the incidence was 15 and 20%, respectively (Hung et al., 2005). A Taiwanese research group conducted further prospective screening of HLA-B*58:01 allele for allopurinol new users; the result showed a preemptive screening of HLA-B*58:01 to be effective in protecting patients from developing allopurinol-induced SCAR (Ko et al., 2015). Although our patients were of the Han Chinese ancestry, similar results were confirmed in Thai, Japanese, and Korean patients, whereas a significant but weaker association was reported in European patients (Kaniwa et al., 2008; Lonjou et al., 2008; Tassaneeyakul et al., 2009; Kang et al., 2011; Cao et al., 2012; Tohkin et al., 2013; Stamp et al., 2016) (**Table 1**). The difference in the strength of association may be due to the frequency of HLA-B*58:01 in different ethnicities. In a previous study, both allopurinol and its metabolite, oxypurinol, induced SCAR development in a dose-dependent manner (Yun et al., 2013). Our recent study also provided a molecular-level understanding of allopurinol-induced SCARs associated with HLA-B*58:01 and activated T cells response without intracellular Ag processing (Lin et al., 2015). Briefly, we showed that Arg97, which is located in the region between the E and C pocket of HLA-B*58:01, is the most probable binding site for drugs like oxypurinol that is consistent with that Arg97 might form a hydrogen bond with oxypurinol in other study (Yun et al., 2014). Furthermore, previous as well as our studies showed that renal impairment was another risk factor for patients with allopurinol-induced SCAR (Yun et al., 2013; Chung et al., 2015). Briefly, renal impairment and delayed clearance of oxypurinol were the poor prognostic factors of allopurinol-SCAR suggesting that delayed excretion of oxypurinol may lead to cumulative toxicity and irreversible deterioration of the prognosis of the disease. A higher mortality rate of allopurinol-SCAR had been observed in chronic kidney disease (CKD) patients, because the use of allopurinol is a common treatment for hyperuricemia in CKD patients (Siu et al., 2006). The results indicated oxypurinol induced T-cell response in a dose and time dependent manner, whereas allopurinol or febuxostat did not. In addition, plasma granulysin levels are associated with renal function impairment, mortality, and delayed clearance of oxypurinol in allopurinol-SCAR. The increases of granulysin and oxypurinol indicate that they may have a vicious cycle on disease progression related to the poor and declining renal function and perhaps the retained oxypurinol induces cytotoxic T cell to release granulysin. These studies suggest that allopurinol use should avoid in patients with renal impairment. These studies suggest that allopurinol use should avoid in patients with renal impairment. Because of the significance of our findings, HLA-B*58:01 genotyping is used clinically to prevent allopurinol-induced SCAR (Hung et al., 2005).

### HLA-Gene Susceptibility to Dapsone-Induced Severe Cutaneous Adverse Reactions

Dapsone (4,4′-diaminodiphenylsulfone) is a sulfone drug with antibiotic and anti-inflammatory effects. It is used frequently to treat infectious conditions such as leprosy and *Pneumocystis jirovecii* pneumonia, and also dermatologic inflammatory diseases including dermatitis herpetiformis and IgA dermatoses. The occurrence of dapsone-induced hypersensitivity was approximately 0.5 to 3.6% in patients after 4- to 6-week treatment (Rao and Lakshmi, 2001). In 2013, a genome-wide-association study reported genetic association of HLA-B*13:01 with hypersensitivity in Chinese population, with the sensitivity of 85.5% and specificity of 85.7% (Zhang et al., 2013) (**Table 1**). Similar results were also reported in Taiwan and Thailand (Tempark et al., 2017; Chen et al., 2018). Recent study indicated that dapsone would fit F-pocket of the antigen-binding site in HLA-B*13:01 and elicit the T-cells response to induce dapsone-hypersensitivity syndrome (DHS) (Watanabe et al., 2017). A study showed that prospective HLA-B*13:01 screening in routine clinical practice would reduce the incidence of dapsone-induced hypersensitivity (Liu et al., 2019) (**Table 2**).

### HLA-Gene Susceptibility to Penicillin Induced Severe Cutaneous Adverse Reactions and Hypersensitivity Reactions

Aminopenicillins is an antimicrobial medicine that is frequently used to treat bacterial infection worldwide. Aminopenicillin and beta-lactam antibiotics may cause hypersensitivity reactions, including SCAR (delayed type) and type 1 hypersensitivity (urticarial or anaphylaxis). A recent study showed HLA-C*04:06, HLA-C*08:01, and HLA-DRB1*04:06 were associated with beta-lactam antibiotics induced delayed type hypersensitivity reactions (MPE, DRESS, and SJS) (Singvijarn et al., 2019). Aminopenicillins may also cause drug-induced liver injury (DILI) and accounts for 10–13% of hospitalizations. A study reported a strong association between HLA and amoxicillin-clavulanate-induced DILI in Europeans (Lucena et al., 2011). The study observed a much higher frequency of DRB1*15:01-DRB5*01:01-DQB1*06:02 haplotype in patients with amoxicillin-clavulanate-induced DILI that was further validated by a study in UK population (Donaldson et al., 2010) (**Table 1**). In addition to aminopenicillins, there has been reported flucloxacillin-induced DILI significantly associated with HLA-B^∗^57:01 (Daly et al., 2009) (**Table 1**). Interestingly, HLA-B*57:01 is a strong risk factor for abacavir hypersensitivity in patients but without liver injury. Also, abacavir‐responsive CD8^+^ clones were not activated by flucloxacillin, and flucloxacillin‐responsive CD8^+^ clones from patients with liver injury and HLA‐B*57:01‐positive volunteers were not stimulated by abacavir (Monshi et al., 2013). Moreover, HLA-B*57:01 was also strongly associated with patients of CBZ-induced SJS/TEN in Europeans (Mockenhaupt et al., 2019). The detailed mechanism of this finding with shared associated HLA allele remains unclear and further investigation was needed. Since the positive predictive value is as low as 0.12%, HLA-B*57:01 is not a good genetic screening marker before new user treated with flucloxacillin (Daly et al., 2009; Yip et al., 2015). Besides of HLA-B*57:01, HLA-B*57:03 is also a major genetic risk factor for the induction of DILI by flucloxacillin (Nicoletti et al., 2019b) (**Table 1**). At present, there is no conclusive reliable HLA allele marker identified for genetic screening before penicillin treatment. In addition to delayed type hypersensitivity reactions, penicillin is one of the most common cause for drug-induced anaphylaxis (type 1 hypersensitivity) (Gonzalez-Estrada and Radojicic, 2015). There have been report that HLA-DRB1*09 was associated with penicillin induced immediate hypersensitive reaction in Chinese while HLA-B*48:01 was associated with beta-lactam penicillin immediate hypersensitive reaction in Thai pediatric population (Yang et al., 2006; Singvijarn et al., 2019).

### HLA Class II and Other Gene Variants for Severe Cutaneous Adverse Reactions

HLA class I plays a predominant role in the induction of SCAR hypersensitivity by T cells. HLA class II genes are associated with SCAR hypersensitivity such as DRESS. For example, Martin AM et al. enrolled nevirapine-exposed cohort to examine the potential-risk factor for hypersensitivity. Patients with nevirapine-induced DRESS were associated with an interaction between HLA-DRB1*0101 and a percentage of CD4^+^ T cells. Their data suggest that HLA-DRB1*0101 and the CD4^+^ status may determine susceptibility to nevirapine hypersensitivity (Martin et al., 2005). Although HLA predisposition is a critical prospective marker for the prevention of drug-induced SCAR, other factors such as the clearance of harmful drugs may be involved in SCAR development. Drug metabolism is a key element in the prevention of body damage from drug toxicity since it reduces drug accumulation. The capacity for drug metabolism may or may not contribute to the incidence of SCAR. Our previous results identified CYP2C9*3 connection to PHT-induced SCAR in Asians patients (Chung et al., 2014; Tassaneeyakul et al., 2016). In addition to CYP2C9*3, concurrently testing CYP2C9*3/HLA‐B*13:01/HLA‐B*15:02/HLA‐B*51:01 can increase the sensitivity and specificity considerably, by 64.7 and 71.9%, respectively. The combined assessment of the risk of HLA and CYP2C9 alleles is useful for predicting PHT-induced SCAR in selected Asian populations (Cheung et al., 2013; Chang et al., 2017; Su et al., 2019). A recent study from Japanese research groups showed that a combination of CYP2C9*3 and HLA‐B*51:01 was associated with PHT-induced hypersensitivity reactions in Japanese population (Hikino et al., 2019). Previously, other study indicated other metabolizing enzyme, CYP2B6, genetic variant involved in nevirapine-induced SJS/TEN. In briefly, Ciccacci C et al. enrolled 27 patients with nevirapine (NVP)-induced SJS/TEN and 78 controls from Mozambique and CYP2B6 had an association with SJS/TEN susceptibility (Ciccacci et al., 2013). Recent study found a strong association between the CYP3A5*3 and antiepileptics-induced hypersensitivity reactions. The study provides evidence that normal CYP3A5*3 activity might be a protective factor to aromatic antiepileptics-induced hypersensitivity reactions in Brazilian subjects (Tanno et al., 2015). Another recent study indicated other genetic variant, the complement factor H-related 4 (CFHR4), to be associated with PHT-induced MPE. Briefly, the author reported an association of a rare variant of the CFHR4 gene in Europeans with phenytoin-induced MPE but not in Han Chinese patients; the results may support the possibility that the complement-system alternative pathway may be involved in phenytoin-induced hypersensitivity in European patients (McCormack et al., 2018). Further, it may be worth to identify non-HLA genetic variants’ association with SCAR hypersensitivity.

## Specific T Cell Receptors for Severe Cutaneous Adverse Reactions

Our previous studies reveal that the infiltration of CTLs into the skin lesions of SJS/TEN leads to further release of inflammatory cytokines and cytotoxic proteins such as granulysin and granzyme B that cause extensive keratinocyte apoptosis (Chung et al., 2008; Chung and Hung, 2012; Wang et al., 2013). In addition to the HLA alleles, recent study also showed a significant role of specific TCRs in the pathogenesis of SCAR (Chung et al., 2015; Pan et al., 2019). Our previous study examined peripheral-blood mononuclear cells (PBMCs) from SCAR patients co-cultured with allopurinol, oxypurinol, or febuxostat; we found that only oxypurinol stimulation resulted in T cells activation with a significant increase in granulysin of cultured samples. Furthermore, analysis of the TCR-repertoire of cells from the skin-blister lesions in allopurinol-SCAR patients and *in vitro*-expanded T cells revealed that blister cells and oxypurinol-expanded T cells possessed preferential TCR-V-β usage and clonal expansion of specific CDR3 (Chung et al., 2015). Recently, we also identify a public TCR composed of a paired TCRα CDR3 (third complementarity-determining region) “VFDNTDKLI” and TCRβ CDR3 “ASSLAGELF” clonotypes from CBZ-SJS/TEN patients of Asians and Europeans. This observation may explain why patients having different HLA alleles become involved in the same hypersensitivity development in different ethnicities. This public TCR shows drug-specificity and phenotype-specificity in an HLA-B*15:02-favored manner. The result from functional assays, co-cultures, and adoptive transfer of TCR-T cells suggests that the drug-specific TCR of CTL may be essential for the immune synapse that mediates CBZ-SJS/TEN (Pan et al., 2019).

## Clinical Implementation and Application

Genetic markers related to drug hypersensitivity have been discovered for many drugs. At present, some important pharmacogenetic markers have been successfully applied in clinical practice. Increasing evidence shows that pharmacogenomics testing is indispensable as an effective preventive method for patients with known high-risk genetic ancestry. For example, cost-effectiveness analysis of HLA-B*58:01 and HLA B*57:01 genotyping before the treatment of allopurinol and abacavir to prevent SCAR has been done, respectively (Hughes et al., 2004; Plumpton et al., 2017; Ke et al., 2017). The population frequency of risk HLA allele, predictive value, costs of the genotyping, and costs of alternative drugs may be the key factors influencing cost-effectiveness. So far, genetic screening is still an important preventable strategy that can keep patients away from SCAR risks.

HLA-B * 57:01 screening of patients before abacavir treatment is widely used in clinical practice. The US FDA, European Medicines Agency (EMA), Canada Health Canada (Sante Canada) (HCSC), and international human-immunodeficiency-virus (HIV)-treatment guidelines recommend HLA-B*57:01 screening before abacavir treatment in routine clinical practice (**Table 2**).

Based on the accumulated scientific evidence, pre-screening for HLA-B*15:02 of patients with Asian background prior to administration of CBZ has been suggested by Taiwan, the US FDA as well as drug-regulatory agencies in other countries (Chen et al., 2011). At present, a preventive genetic test of HLA-B*15:02 for CBZ new users has been supported by the national health insurance in Taiwan, Hong Kong, Singapore, Thailand, and mainland China (Tiamkao et al., 2013; Chen et al., 2014) (**Table 2**). In addition to HLA-B*15:02, the Taiwan and U.S. FDA have mentioned HLA-A*31:01 is another risk genetic marker for CBZ-induced hypersensitivity reactions and request HLA-A*31:01 genetic testing before the use of CBZ in patients of Asian descent. HCSC recommends HLA-A*31:01 genetic testing before the use of CBZ (**Table 2**). Moreover, U.S. FDA suggests HLA-B*15:02 testing recommended before patients treated with oxcarbazepine.

Another typical pharmacogenomics translation to clinical utility is the use of HLA-B*58:01 to protect patients from the risk of allopurinol-induced SCAR. Owing to the significant association of HLA-B*58:01 with allopurinol-induced SCAR, the American College of Rheumatology (ACR) guidelines for the management of gout recommended in 2012 HLA-B*58:01 testing prior to allopurinol administration (Khanna et al., 2012). A number of medical centers in Taiwan, Hong Kong, Thailand, and mainland China provide such prescreening; this is beneficial for subjects at risk of allopurinol-associated potentially fatal hypersensitivity reaction (Ke et al., 2019) (**Table 2**). At present, the preventive test of HLA-B*58:01 for allopurinol has been supported by the national health insurance in Korea and China (**Table 2**).

For prevention of dapsone hypersensitivity, HLA-B*13:01 preventive test has been provided for new dapsone users in leprosy centers of China. US FDA and Canada Health Canada have labeled the G6PD deficiency for dapsone related hemolysis, but not HLA-B*13:01 yet for dapsone hypersensitivity. For prevention of PHT-SCAR, an ongoing clinical trial of preventive testing for CYP2C9*3 and the risk HLA alleles is being conducted in Taiwan and China. In addition, HCSC also recommends HLA-B*15:02 genetic testing before the use of PHT (**Table 2**).

## Future Perspectives and Conclusions

Although the incidence of SCAR is low, the condition is considered life-threatening because of the associated high death rate and a huge impact on healthcare systems worldwide. Newer genetic association of drug hypersensitivity will be explored base on their pathogenesis. However, the translation from these research findings into clinical application, several points require stringent persuasion. First, non-genetic factors, such as hepatic or renal impairment for drug metabolism, involved in drugs hypersensitivity. For example, high concentration of metabolites oxypurinol have strong association with granulysin of activation T cells in patients with SCAR hypersensitivity (Chung et al., 2015). Secondly, there are many factors, includes drugs, HLA alleles as well as TCR, involved in SCAR hypersensitivity. For example, drug may affect TCR arrangement, not HLA alleles, to form different TCR clones and further result in general T cells activation. Thirdly, the phenotypic diagnostic criteria (MPE, AGEP, SJS/TEN, and DHS) of individual drug hypersensitivity reactions must be clearly delineated and the genetic association of each drug must be explored. Fourthly, the genetic susceptibility of individual drugs should be reassessed based on ethnic background. Fifthly, in order to increase the statistical intensity of these studies, a large number of cases should be included. Given the rarity of hypersensitivity of certain drugs, this may require multi-center or even cross-country research. The final point to consider is the availability and cost-effectiveness of screening tests. In this regard, simpler design, faster and cheaper testing are essential. In future, further studies and clinical trials on the therapeutics as well as cost-effective aspects are needed so as to develop more strategies for SCAR prevention and management.

## Author Contributions

S-IH and C-BC contributed to the conception. C-JC and C-BC writing of the manuscript. W-HC reviewed the manuscript. All authors contributed to the article and approved the submitted version.

## Conflict of Interest

The authors declare that the research was conducted in the absence of any commercial or financial relationships that could be construed as a potential conflict of interest.
